# Notch signaling contributes to the maintenance of both normal neural stem cells and patient-derived glioma stem cells

**DOI:** 10.1186/1471-2407-11-82

**Published:** 2011-02-22

**Authors:** Yi-Yang Hu, Min-Hua Zheng, Gang Cheng, Liang Li, Liang Liang, Fang Gao, Ya-Ning Wei, Luo-An Fu, Hua Han

**Affiliations:** 1 State Key Laboratory of Cancer Biology, Department of Medical Genetics and Developmental Biology, Fourth Military Medical University, Chang-Le Xi Street #17, Xi'an 710032, PR China; 2 Department of Neurosurgery, Xijing Hospital, Fourth Military Medical University, Chang-Le Xi Street #17, Xi'an 710032, PR China

## Abstract

**Background:**

Cancer stem cells (CSCs) play an important role in the development and recurrence of malignant tumors including glioma. Notch signaling, an evolutionarily conserved pathway mediating direct cell-cell interaction, has been shown to regulate neural stem cells (NSCs) and glioma stem cells (GSCs) in normal neurogenesis and pathological carcinogenesis, respectively. However, how Notch signaling regulates the proliferation and differentiation of GSCs has not been well elucidated.

**Methods:**

We isolated and cultivate human GSCs from glioma patient specimens. Then on parallel comparison with NSCs, we inhibited Notch signaling using γ-secretase inhibitors (GSI) and assessed the potential functions of Notch signaling in human GSCs.

**Results:**

Similar to the GSI-treated NSCs, the number of the primary and secondary tumor spheres from GSI-treated GSCs decreased significantly, suggesting that the proliferation and self-renewal ability of GSI-treated GSCs were attenuated. GSI-treated GSCs showed increased differentiation into mature neural cell types in differentiation medium, similar to GSI-treated NSCs. Next, we found that GSI-treated tumor spheres were composed of more intermediate progenitors instead of CSCs, compared with the controls. Interestingly, although inhibition of Notch signaling decreased the ratio of proliferating NSCs in long term culture, we found that the ratio of G2+M phase-GSCs were almost undisturbed on GSI treatment within 72 h.

**Conclusions:**

These data indicate that like NSCs, Notch signaling maintains the patient-derived GSCs by promoting their self-renewal and inhibiting their differentiation, and support that Notch signal inhibitor GSI might be a prosperous candidate of the treatment targeting CSCs for gliomas, however, with GSI-resistance at the early stage of GSCs cell cycle.

## Background

Glioma, the most common tumor of the central nervous system (CNS), frequently leads to death. Glioma is derived from brain glial tissue and comprises several diverse tumor forms and grades. Treatment of malignant gliomas is often palliative due to their infiltrating nature and high recurrence. Despite advances in surgery, chemotherapy and radiation gradually result in therapy-resistance. However, genetic events that lead to gliomas are mostly unknown.

Recent researches highlight the importance of cancer-initiating cells in the malignancy of gliomas [1-3]. These cells have been referred to as glioma stem cells (GSC), as they share similarities to normal neural stem cells (NSCs) in the brain. There is increasing evidence that malignant gliomas arise from and contain these minority tumor cells with stem cell-like properties. This subpopulation of tumor cells with the potential for self-renewal and multi-lineage differentiation that recapitulates the phenotype of the original glioma [4-8], plays an important role in glioma initiation, growth, and recurrence. Eliminating GSCs from the bulk tumor mass seems to be a prosperous therapeutic strategy [9,10]. Therefore, it is extremely important to understand the signal pathways that contribute to the formation and maintenance of GSCs.

A number of signal pathways are involved in the formation and maintenance of stem cells, many of which are closely conserved across species. Notch signaling, an evolutionarily conserved pathway mediating direct cell-cell interaction and signaling, plays a pivotal role in the maintenance of NSCs [11]. The functions of the Notch pathway in cancer formation have been gradually established, and recent data have also implicated a role for Notch signaling in GSCs [12]. Notch is a family of hetero-dimeric transmembrane receptors composed of an extracellular domain responsible for ligand recognition, a transmembrane domain, and an intracellular domain involved in transcriptional regulation. When Notch receptor is triggered by the ligands on the neighboring cells, the intracellular domain of the Notch receptor (NICD) is released from the membrane, after successive proteolytic cleavages by the γ-secretase complex [13,14]. NICD then translocates into the nucleus and associates with the transcription factor RBP-J, the DNA recombination signal binding protein-Jκ. The NICD-RBP-J complex further recruits other co-activators, and activates the expression of downstream genes associated with cell proliferation, differentiation and apoptosis [15]. It is believed that γ-secretase inhibitors (GSI) decrease the activity of Notch signaling and slow the growth of Notch-dependent tumors such as medulloblastoma [12].

Rapid proliferation, self-renewal ability and multipotential differentiation are the hallmarks of both normal NSCs and GSCs. Similarities in the growth characteristics and gene expression patterns of normal NSCs and brain tumor CSCs suggest that pathways important for NSCs are probable targets for eliminating brain tumor CSCs. The RBP-J-mediated canonical Notch pathway plays several significant roles in the maintenance and differentiation of NSCs [16-18]. During embryogenesis, Notch signaling is required to maintain all NSC populations, and to repress the differentiation of NSCs into intermediate neural progenitors (INPs) in vivo [19-21]. Along with later development, Notch signal commits NSCs to an astroglia fate, while repressing neuronal differentiation [22]. In adult, Notch signaling modulates cell cycle in order to ensure brain-derived NSCs retain their self-renewal property [23].

Increasing evidence has shown that there is a link between tumorigenesis and aberrantly activated Notch signaling [24,25]. Notch1 and its ligands, Dll1 and Jagged1, were overexpressed in many glioma cell lines and primary human gliomas. When the expression of Notch1, Dll1 or Jagged1 was down-regulated by RNA interference, apoptosis and proliferation inhibition in multiple glioma cell lines were induced [26]. Depletion of Hey1, a member of Hes-related family downstream effectors of Notch signaling, by RNA interference also reduces proliferation of glioblastoma cells in tissue culture [27]. Moreover, the blockade of Notch signaling directly caused cell cycle exit, apoptosis, differentiation, and reduced the CD133-positive cells in medulloblastoma and glioblastoma cell lines while Notch activation enhances the expression of Nestin, promotes cell proliferation and the formation of NSC-like colonies and plays a contributing role in the brain tumor stem cells [28-30]. However, the exact roles of Notch signaling in the proliferation and differentiation of patient-derived GSCs have not been clearly elucidated.

In this study, we explore the roles of Notch signaling in patient-derived GSCs with parallel analysis of normal NSCs by using GSI-mediated inhibition of Notch signaling in vitro. The results showed that when Notch signaling was inhibited, the proliferation and self-renewal ability of GSCs from human primary gliomas were attenuated. In addition, the blockade of Notch signaling in GSCs increased their differentiation into the downstream neural cell types, and promoted their conversion from stem cells into INP-like cells. Interestingly, although inhibition of Notch signaling definitely decreased the proliferating GSCs in long term culture, we found that the percentage of G2+M phase-GSCs were almost undisturbed at the initial stage of GSI treatment. To summarize, our results suggested that Notch signaling maintained GSCs by promoting their self-renewal and inhibiting their differentiation into INP-like cells, and supported that Notch signal inhibitors might be prosperous candidates of the treatments targeting CSCs for gliomas.

## Methods

### Glioma samples

Glioma tissues were obtained from 9 adult patients with pathologically diagnosed grade 2 to grade 4 gliomas, at the Department of Neurosurgery in Xijing Hospital, Fourth Military Medical University, under the guidance from the Medical Ethnic Committee of the Fourth Military Medical University. The summary of the patient population is outlined in Additional file 1: Table S1.

### Neurosphere culture

Neurosphere cultures were performed as described previously with some modifications [21]. Briefly, for the culture of NSCs, the brains from embryonic (E) day 12.5 C57BL/6 mice were dissected under a stereomicroscope. And for the culture of GSCs, tissues from patient specimen were acutely minced after sampling. The tissues were then washed, mechanically dissociated by repetitive pipette. Single cells were primarily plated in serum-free Dulbecco's modified Eagle's medium (DMEDM)/F12 medium containing 20 ng/ml basic fibroblast growth factor (bFGF, human recombinant, Sigma), 20 ng/ml epidermal growth factor (EGF, mouse submaxillary), the B-27 (1:50, GIBCO), penicillin (100 U/ml) and streptomycin (0.1 mg/ml). Cells were cultured at a density of 1 × 10^5 ^cell/ml in 24-well plates (0.5 ml/well), and were fed every 3 days by adding fresh medium supplemented with GSI or DMSO with indicated concentrations. Animal experiments were reviewed and approved by the Animal Experiment Administration Committee of the Fourth Military Medical University.

### Neurosphere assays

After 7 days from primary culture the numbers of primary spheres were counted under a microscope (Additional file 1: Figure S2) [21]. And for the expression of target genes, neurospheres were harvested on the 5th day of culture for RNA extraction, cDNA synthesis, and real-time reverse transcription-polymerase chain reaction (RT-PCR). Primary neurospheres were harvested and dissociated mechanically into single cell suspensions, and were replated at 1 × 10^5 ^cells/ml in 24-well plates. Cells were then cultured for another 7 days until secondary spheres formed [31], which were quantified by counting. On the 7th day of primary culture, neurospheres were plated onto poly-D-lysine (Sigma) coated glass cover slips in DMEM/F12 containing 10% fetal bovine serum (FBS) for another 7 days. On the third day of differentiation, neurospheres were photomicrographed and their neurites were counted and measured, then on the 7th day of differentiation culture, immunofluorescence staining was performed as described below.

### Immunofluorescence

Undifferentiated neurospheres were plated onto poly-D-lysine coated glass cover slips in serum-free medium for 4 h. Then cells were directly fixed in 4% paraformaldehyde at 4°C for 10 min, and incubated with primary antibodies overnight at 4°C, followed by species-specific secondary antibodies. Samples were visualized under fluorescence microscope (FV-1000, Olympus, Japan). Immunofluorescence for differentiated neurospheres was performed in a similar way. Cells were additionally counterstained with Hoechst. Primary antibodies used included rabbit anti-Nestin serum (1:200, Sigma), rabbit anti-glial fibrillary acidic protein (GFAP, 1:200, Sigma), mouse anti-mitogen-activated protein 2 (MAP2, 1:200, Sigma). FITC-conjugated goat anti-mouse IgG and Cy3-conjugated goat anti-rabbit IgG (1:400, Jackson ImmunoResearch) were used as the secondary antibodies.

### Quantitative RT-PCR

Total RNA of neurospheres was isolated using the Trizol reagent (Invitrogen). cDNA was synthesized and was used for real-time PCR with a kit (SYBR Premix EX Taq, Takara, Kyoto, Japan) and the ABI PRISM 7300 real-time PCR system, with human GAPDH and mouse β-actin as the reference controls. Primers used for real-time PCR were summarized in Additional file 1: Table S3.

### DNA content analysis

Spheres were dissociated mechanically into single cell suspensions in the culture medium. Cells were then washed and resuspended in PBS, and were fixed with ethanol at room temperature for 20 min. Cells were resuspended in PBS containing 50 μg/ml of propidium iodide and 0.1 mg/ml RNase A for 10 min, and were analyzed for ploidy using a flow cytometry (BD Biosciences). Data analysis was performed using the CellQuest software (BD Biosciences).

### Statistics

Independent cultures from at least three samples were used for each experiment (Additional file 1: Table S2). For immunofluorescence, cells were counted by Image-ProPlus 6.0, and only cell bodies that were labeled with immunoreactivity were included. Proportions of immunoreactive cells in the total population of cultured cells revealed by Hoechst staining were calculated, and at least 5 microscopic fields per specimen were selected. For neurite analysis, neurites of 30 neurospheres from each culture in the presence of GSI or DMSO were measured. The total numbers of neurites per tumor spheres were counted via photomicrographs taken by a phase contrast microscopy, and the average of the length of neuritis per tumor spheres were measured by Image-ProPlus 6.0. Each experiment was repeated for at least three times. Data were expressed as mean ± s.e.m, and the difference between the two groups was analyzed with the Student's t-test, with *P *< 0.05 as statistically significant.

## Results

### Formation of neurosphere-like colonies from primary glioma specimens

Nine specimens of gliomas were used in the current studies, including 3 oligoastrocytomas, 3 oligodendrogliomas, 2 astrocytomas, and 1 glioblastoma, and the specimens were graded according to the WHO grading scheme (Additional file 1: Table S1).

**Figure 1:**
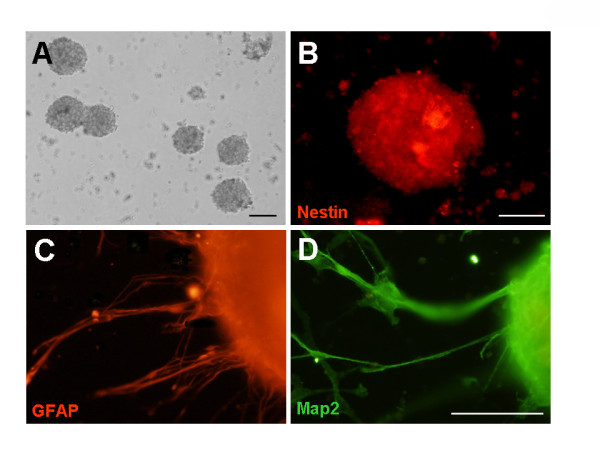
**Patient glioma-derived stem cells have the ability to form neurosphere-like colonies and gave rise to the downstream neural cell types of NSCs**. (A) Photomicrographs of typical primary tumor spheres from one glioma tissue at 72 h after plating. (B) Undifferentiated primary tumor spheres expressed high levels of Nestin (red), a marker of NSCs. (C, D) The tumor spheres-derived from human glioma were cultured in differentiation conditional medium for 7 days, and differentiated into neural cells expressing specific molecular markers of GFAP (C, red) and MAP2 (D, green). Scale bar, 100 μm in A, and 50 μm in BCD.

Tumor tissues were dissociated mechanically into a single cell suspension and were cultured in serum-free DMEM/F12 medium supplemented with EGF and bFGF. Seven primary gliomas of the nine gave rise to proliferating tumor spheres. Regardless of pathological subtype and grade, neurosphere-like clusters, or tumor spheres, first appeared within 72 h of primary culture and increased their numbers and diameters quickly during 7 days after the onset of the culture (Figure 1A). In order to estimate whether these tumor spheres showed NSC properties, we stained the tumor spheres from patients with anti-Nestin antibody. The result showed that these tumor spheres expressed Nestin, a marker of NSCs (Figure 1B). The multipotency of these human glioma cell-derived tumor spheres was confirmed by differentiation assay in vitro. We estimated the differentiation capacity of tumor spheres in differentiating conditions by examining the types of molecular markers expressed by neurons and glial cells. We observed that these cells could differentiate into GFAP-positive astrocyte- and MAP2-positive neuron-like cells (Figure 1C, 1D). In addition, a local recurrence tumor also could produce tumor spheres in growth medium (data not shown). Tumor spheres could be passed at least for five generations by mechanical dissociation and their stemness and multipotency could be maintained in serum-free medium supplemented with growth factors for at least one month.

### Blockade of Notch signaling attenuates the proliferation and self-renewal ability and promotes differentiation of normal NSCs

**Figure 2:**
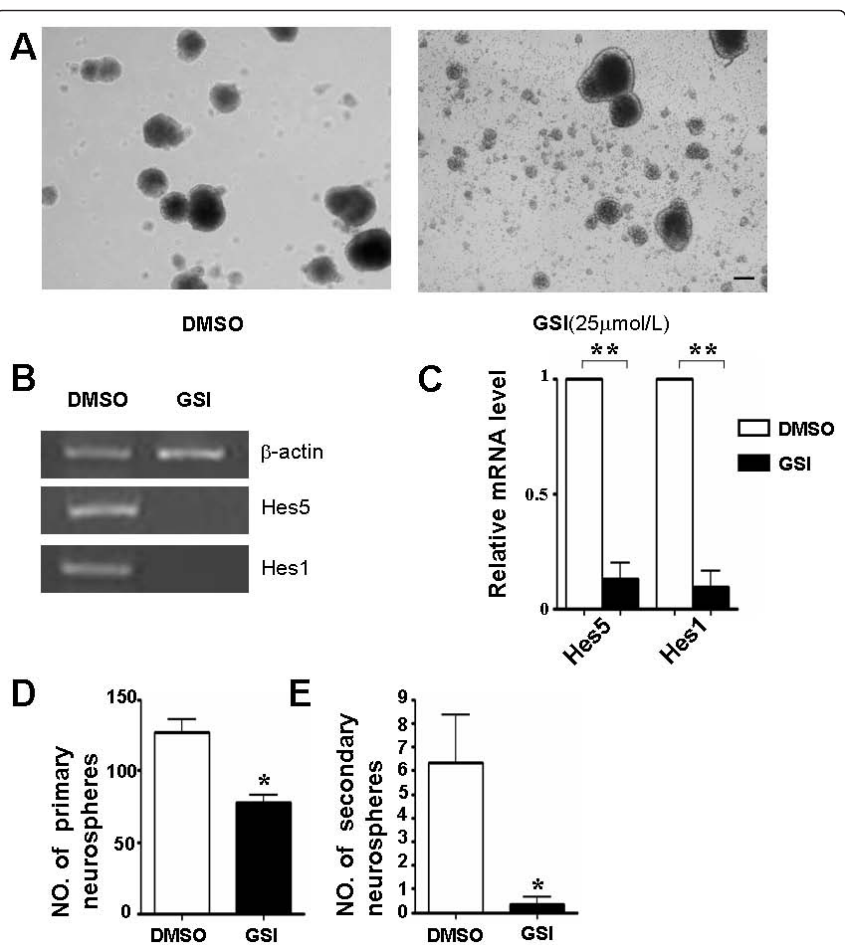
**Blockade of Notch signaling attenuates proliferation and self-renewal ability of normal mouse NSCs**. (A) Photomicrographs of neurospheres derived from E12.5 mouse brain at 72 h after primary culture, with GSI or DMSO supplemented. (B, C) Total RNA was prepared from neurospheres on the 5th day of GSI or DMSO treatment. And the expressions of Hes1 and Hes5 were measured by RT-PCR (B) and Real-time PCR (C), with β-actin as the reference control (n = 3, Hes5, *P *= 0.006, Hes1, *P *= 0.006). (D, E) Equal number of cells (1 × 10^5^/ml) were plated in the growth medium, and the number of primary (n = 3, *P *= 0.010) (D) and secondary (n = 3, *P *= 0.043) (E) neurospheres were counted 7 days after plating. *, *P *< 0.05, **, *P *< 0.01.

Stem cell-like cells in brain tumors share many similarities with normal neural stem/progenitor cells and may require Notch signal for their survival and growth. In vitro, NSCs proliferate and form clonal spheres referred to as neurospheres. GSI reduced the proliferation of mouse embryonic brain-derived NSCs in a dose-dependent fashion (Additional file 1: Figure S1). The number of neurospheres was decreased in the presence of GSI, compared with the control treated with DMSO (Figure 2A). In order to confirm that GSI effectively blocked Notch signaling in NSCs in our culture system, we test the expression of Hes1 and Hes5, both of which are downstream molecules of the Notch signaling [12]. Total RNA was prepared from neurospheres on the fifth day of 25 μmol/L GSI treatment and was used for RT-PCR. The expression of Hes1 and Hes5 decreased remarkably in NSCs, suggesting that GSI at this concentration could inhibit Notch signaling effectively (Figure 2B, 2C). We quantitatively analyzed the number of primary neurospheres in the presence of GSI, and found that there was a significant decrease in the number of neurospheres upon GSI treatment at 25 μmol/L (Figure 2D). In order to determine the possible effect of GSI on the NSCs self-renewal ability, we harvested the spheres and dissociated them into a single cell suspension by soft pipeting. When replated in the presence of GSI, the number of secondary neurospheres significantly decreased after 7 days culture (Figure 2E). These results suggested that the proliferation of NSCs was slowed by inhibiting Notch signaling and the self-renewal ability, a key NSC behavior, was at least partially depleted.

**Figure 3:**
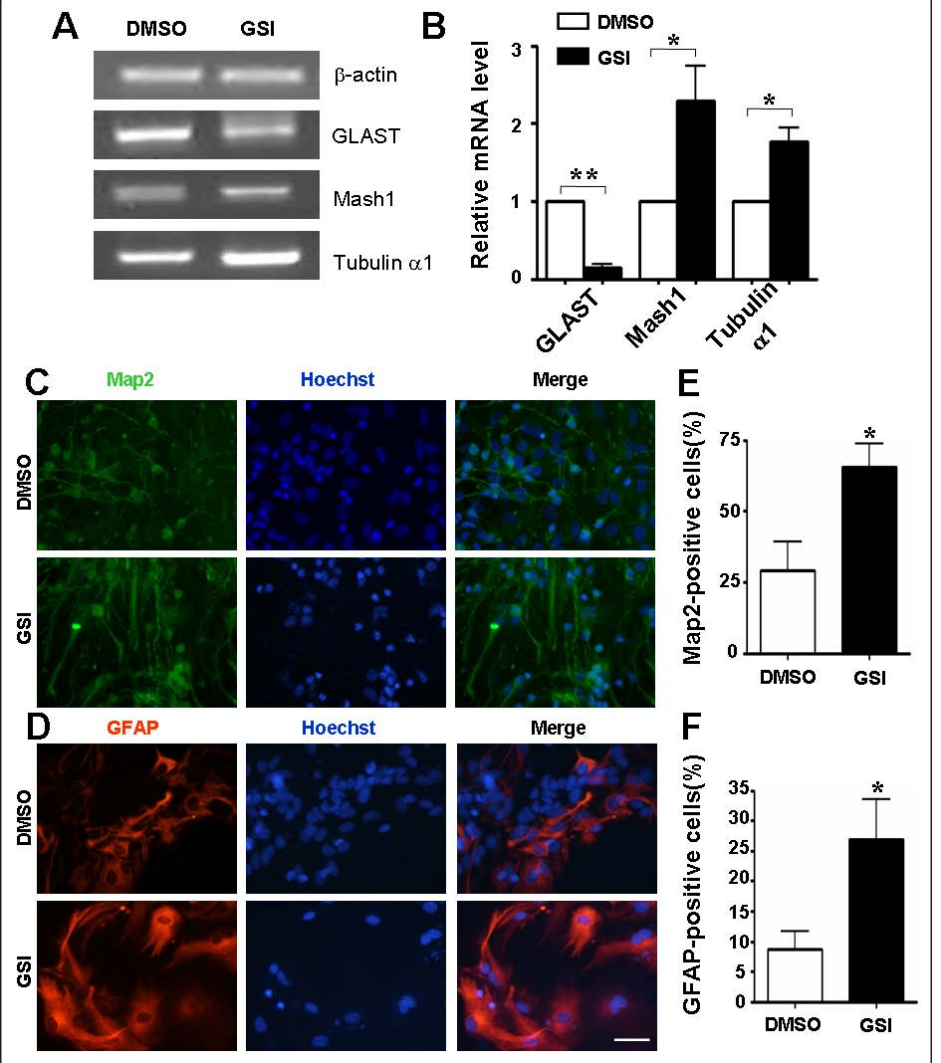
**Blockade of Notch signaling promotes the differentiation of normal mouse NSCs into INPs and downstream neural cell types**. (A, B) Total RNA was prepared from GSI or DMSO treated neurospheres derived from E12.5 mouse brain on the 5th day of culture. And the expressions of Glast, Mash1 and Tubulin α1 were measured by RT-PCR (A) and Real-time PCR (B), with β-actin as the reference control (n = 3, GLAST, *P *= 0.003, Mash1, *P *= 0.043, Tubulin α1, *P *= 0.046). (C, D) Immunofluorescence. Differentiated NSCs were stained with anti-GFAP, or anti-MAP2 antibodies after cultured on cover slips in differentiation conditional medium for 7 days. Stained samples were examined under a fluorescence microscope. (E, F) Quantification and comparison of neurons (MAP2^+^) or astrocytes (GFAP^+^) in GSI-treated and control NSCs. Cells were counterstained with Hoechst, to permit counting of cell nuclei in at least 5 microscopic fields per specimen (n = 3, E, *P *= 0.021, F, *P *= 0.031). *, *P *< 0.05, **, *P *< 0.01. Scale bar, 50 μm for C and D.

Notch signaling has been shown to inhibit the differentiation of NSCs to INPs [21]. In our study, we tested the expression of molecular markers of INPs in primary neurospheres treated with GSI or DMSO. Quantitative RT-PCR showed that the mRNA levels of Glast, which is indicative of the frequency of NSCs, were decreased, while that of Mash1 and Tubulin α1, both of which are markers of INPs, were increased (Figure 3A, 3B). These results indicated an augmented differentiation from NSCs into INPs upon the blockade of Notch signaling by GSI.

To further study the effect of inhibiting Notch signaling on NSC differentiation, we used the neurosphere differentiation assay in vitro. When spheres were cultured adherently on poly-D-lysine coated glass cover slips without growth factors, they began to differentiate into cells bearing specific markers of neurons and astrocytes. We quantitatively compared the cell types produced by neurospheres in the GSI-containing medium with that of the control. All of the neurospheres gave rise to cells with the molecular markers of neurons or astrocytes (Figure 3C, 3D). However, the percentage of MAP2^+ ^cells increased significantly in the presence of GSI, from 29.0 ± 10.4% to 66.5 ± 8.4%, and the percentage of GFAP^+ ^cells in GSI-treated neurospheres was elevated from 8.7 ± 3.0% to 26.9 ± 6.6% (Figure 3E, 3F). These results suggested that inhibiting Notch signaling in NSCs leads to an increase in the number of differentiated cells.

### Decreased proliferation and self-renewal ability of GSCs upon GSI treatment

**Figure 4:**
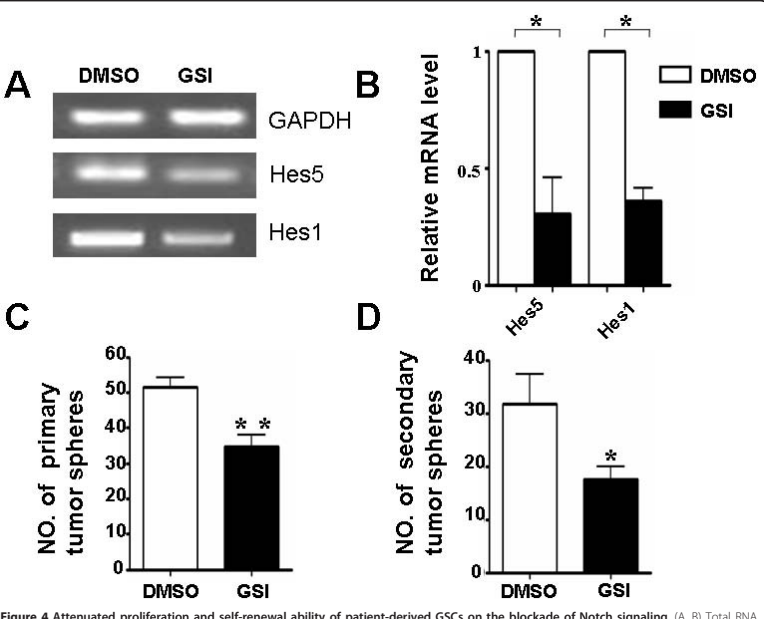
**Attenuated proliferation and self-renewal ability of patient-derived GSCs on the blockade of Notch signaling**. (A, B) Total RNA was prepared from primary tumor spheres on the 5th day in the presence of GSI or DMSO. And the expressions of Hes5 and Hes1 were measured by RT-PCR (A) and Real-time PCR (B), with human GAPDH as the reference control (n = 5, Hes5, *P *= 0.046, Hes1, *P *= 0.002). (C, D) Equal number of cells (1 × 10^5^/ml) form brain tumor tissues were plated in the growth medium, the number of primary (n = 4, *P *= 0.008) (C) and secondary (n = 4, *P *= 0.041) (D) tumor spheres were counted 7 days after plating. *, *P *< 0.05, **, *P *< 0.01.

Although Notch signaling has been shown to play critical roles in the maintenance of normal NSCs, whether this signaling might be involved in tumor stem cells is not fully clear. To determine whether Notch signaling activity was required during growth of GSCs, we investigate the effect of GSI on proliferation and self-renewal of GSCs. After Notch signaling was inhibited in GSCs by GSI treatment at 25 μmol/L, the expressions of Hes5 and Hes1, the specific and direct downstream targets of the Notch/RBP-J transcription complex were identified by RT-PCR and real-time PCR as described previously. After 5 days of GSI treatment, Hes5 and Hes1 expression markedly decreased (Figure 4A, 4B), and no obvious cell death was observed, indicating no effect on cell viability (data not shown). These results indicated that Notch signaling was efficiently blocked by GSI treatment in GSCs.

Next, we quantitatively compared the proliferation and self-renewal ability of GSI-treated GSCs with that of the controls. The number of the primary tumor spheres in the presence of GSI decreased significantly, from 51.5 ± 2.8 to 34.8 ± 3.3 (Figure 4C). Self-renewal ability of the tumor spheres was assayed by dissociating and replating the primary tumor spheres. Our results showed that GSI-treated GSCs generated a decreased number of secondary tumor spheres (17.5 ± 2.3), than the number of controls (31.7 ± 5.6) (Figure 4D). These results showed that the proliferation and self-renewal ability of GSCs also could be attenuated by inhibiting Notch signaling.

### Blockade of Notch signaling promotes the differentiation of GSCs

**Figure 5:**
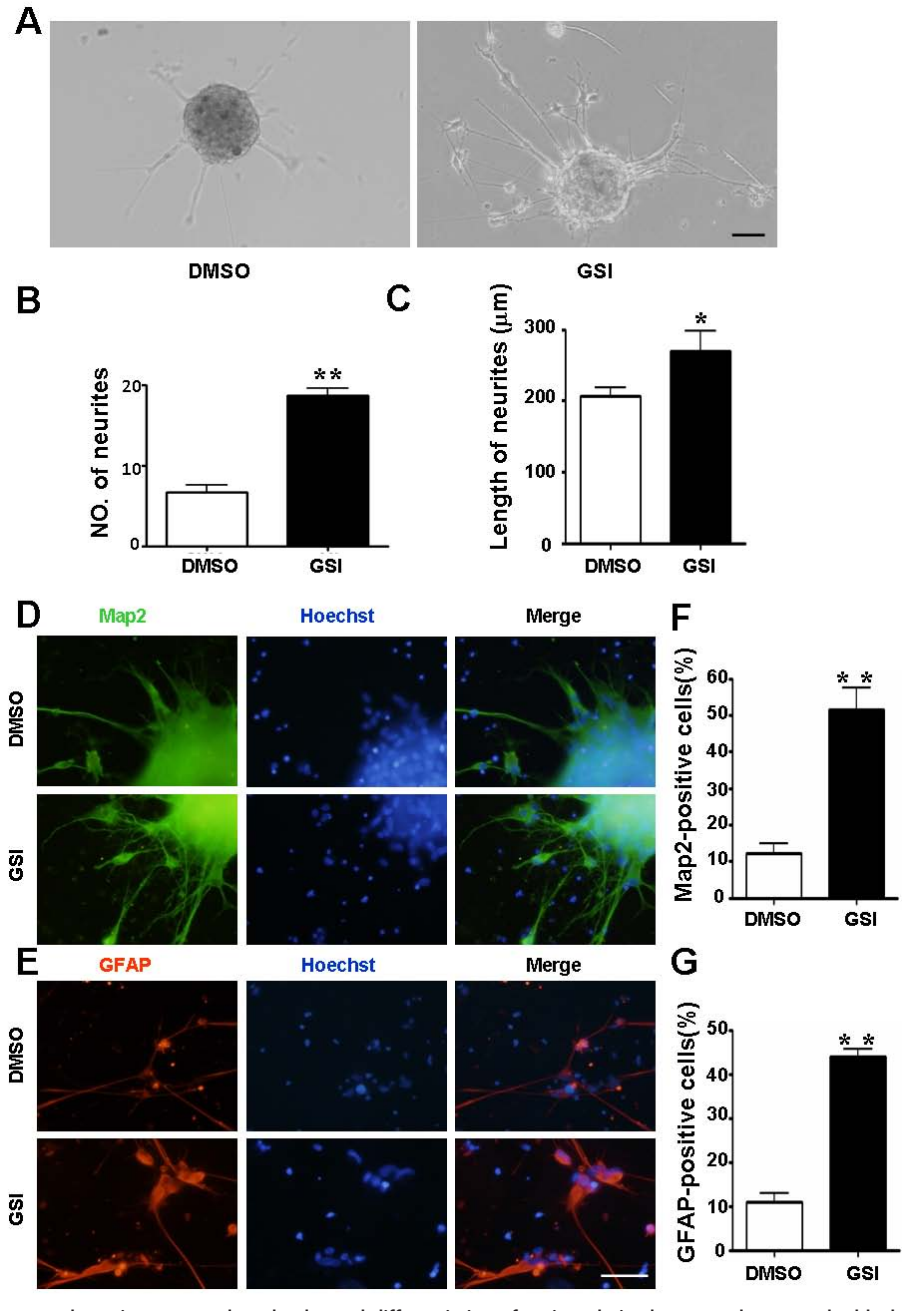
**Augmented neurite outgrowth and enhanced differentiation of patient-derived tumor spheres on the blockade of Notch signaling**. (A) Photomicrographs of differentiated tumor spheres at 72 h after plated in differentiation conditional medium supplemented with GSI or DMSO. (B, C) Comparison of neurites number (n = 3, *P *< 0.001) (B) and length (n = 3, *P *= 0.041) (C) between tumor spheres in the presence of GSI and DMSO. (D, E) Immunofluorescence. Differentiated tumor spheres were stained with anti-GFAP, or anti-MAP2 antibodies after cultured on cover slips in differentiation conditional medium for 7 days. Stained samples were examined under a fluorescence microscope. (F, G) Quantification and comparison of the percentages of neurons (MAP2^+^) (n = 3, *P *< 0.001) (F) or astrocytes (GFAP^+^) (n = 3, *P *< 0.001) (G) in the total cell number revealed by Hoechst counterstaining, between GSI-treated and control GSCs. Scale bar, 100 μm for A, and 50 μm for D and E. *, *P *< 0.05, **, *P *< 0.01.

The previous result indicated that inhibiting Notch signaling promotes the normal NSCs to differentiate into neurons and astrocytes, both of which are the downstream neural cell types of NSCs. Therefore, we investigated whether the GSI treatment promoted GSCs differentiation. Interestingly, after 3 days, approximately 18.7 ± 0.9 neurites grew out from each tumor spheres cultured in the medium with GSI, compared to only 6.7 ± 0.9 from that cultured with DMSO. Meanwhile, the average length of neurites increased from 206.0 ± 13.1 μm in tumor spheres culture with DMSO to 269.7 ± 28.4 μm in GSI-treated tumor spheres (Figure 5B, 5C). In order to further confirm whether these cells are the downstream neural cell types, immunofluorescence was performed on differentiated primary GSCs using the specific markers of neurons and astrocytes on the 7th day in differentiating conditional medium (Figure 5D, 5E). We quantitatively compared the cell types produced by neurospheres in the GSI-treated group with that of the control. The percentages of MAP2^+ ^cells and GFAP^+ ^cells increased significantly, as high as 51.6 ± 6.1% and 44.0 ± 1.7%, respectively (Figure 5F, 5G). These results suggest that inhibiting Notch signaling also promotes the differentiation of GSCs.

### Blockade of Notch signaling promotes the conversion of GSCs to INP-like cells

**Figure 6:**
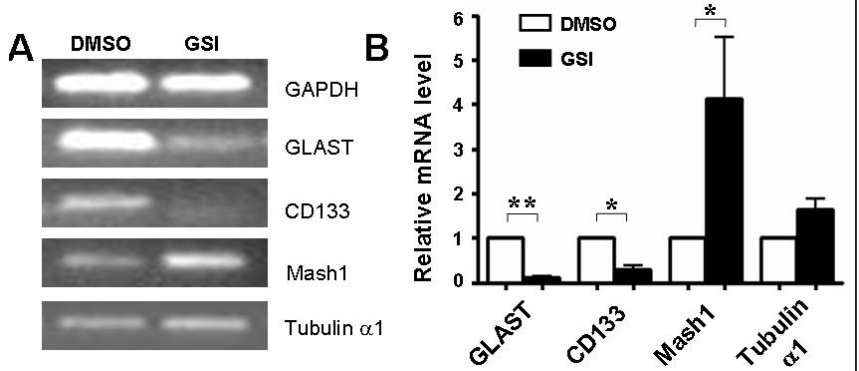
**GSI-treated primary tumor spheres show similar gene expression profile of INPs**. (A, B) cDNA was prepared from total RNA isolated from primary tumor spheres, treated with GSI or DMSO for 5 days respectively, and the expressions of GLAST (*P *= 0.002), CD133 (*P *= 0.015), Mash1 (*P *= 0.050) and Tubulin α1 (*P *= 0.116), were measured by RT-PCR (A) and Real-time PCR (n = 5) (B), with GAPDH as a reference control. *, *P *< 0.05, **, *P *< 0.01.

The previous report indicated that blockade of Notch signaling in the CNS increased the frequency of INPs in vivo [21]. Precocious differentiation of NSCs into INPs might exhaust the NSC pool. Therefore, we investigated the effect of inhibiting Notch signaling on the frequency of GSCs and INP-like cells in glioma specimen. In an attempt to distinguish GSCs and INP-like tumor cells, we examined the expression of several markers that could distinguish NSCs from INPs by quantitive RT-PCR [20,21]. Compared with the controls, the primary tumor spheres in the presence of GSI expressed lower Glast and CD133, which are indicative of the frequency of NSCs and GSCs. In contrast, Mash1 was highly expressed in GSI-treated tumor spheres (Figure 6A, 6B), although the expression level of another INP marker, Tubulin α1 was comparable between the GSI-treated tumor spheres and that of control. Altogether, these results suggested that blockade of Notch signaling may promote the conversion of GSCs to INP-like tumor cells.

### GSCs show resistance to GSI treatment compared with NSCs

**Figure 7:**
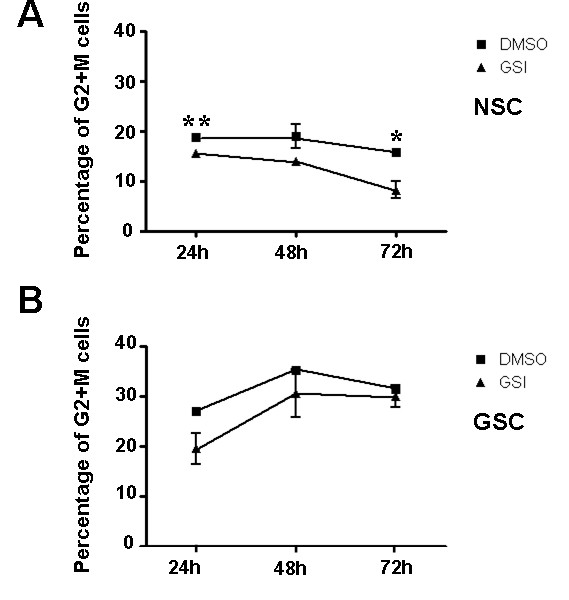
**Different effects of GSI-treatment on the cell cycle of NSCs or GSCs**. (A, B) Comparisons of cell cycle between NSCs (A) and GSCs (B) in the presence of GSI or DMSO at 24 h, 48 h, 72 h using flow cytometry. Data represent as mean ± SD from three independent experiments. (n = 3, 24 h, *P *= 0.006, 48 h, *P *= 0.013). *, *P *< 0.05, **, *P *< 0.01.

To gain further perspective on the dynamics of cellular proliferation accompanying differentiation, we treated NSCs and tumor spheres at a series of time points following GSI treatment with propidium iodide and examined cell cycle via FACS analysis. Compared with the controls, nearly 15.5 ± 0.5% of the NSCs treated with GSI for 24 h are in the G2+M phase, and then sharply decreased to less than 8.2 ± 1.7% at 72 h (Figure 7A). In contrast, the ratio of GSCs in the G2+M phase were slightly elevated at 48 h, and then declined insignificantly at 72 h (Figure 7B). The result showed that GSI treatment significantly reduced the ratio of the G2+M phase NSCs, but there is no obvious effect on the cell cycle of GSCs. Therefore, NSCs are more sensitive to GSI, while GSCs display a certain degree of resistance to GSI at the early stage of the treatment.

## Discussion

Tumor stem cells such as GSCs have been considered as a novel target for the therapy of the malignant tumors, because these cells are supposed to play an important role in tumor initiation, growth, and recurrence [4]. Similarities in the growth characteristics and gene expression patterns of normal NSCs and GSCs suggest that pathways important for NSCs are probable targets for oncogenic brain tumor stem cells. In the present study, 1) we isolated GSCs from the human glioma tissues; 2) Like NSCs, these cells had the ability to form spheres in serum-free medium supplemented with growth factors and differentiated into downstream neural cell-like cells; 3) By GSI treatment, the number of GSCs-derived primary neurospheres and secondary neurospheres were markedly reduced compared with those treated with DMSO, indicating that in the long term culture (7-14 days), the proliferation and self-renewal ability of GSCs was ultimately reduced, upon the blocking of Notch signaling; 4) However, within 72 h culture, GSCs showed a certain degree of GSI-resistance, with undisturbed proliferation ability upon GSI treatment; 5) In addition, we showed that on blocking Notch signaling, GSCs are much biased to differentiate into INP-like cells, and ultimately neurons and glial cells in vitro. All these results suggest a promising preclinical application of Notch signaling antagonist (e.g., GSI)-based CSCs-targeting therapy in malignant glioma patients.

### The frequency of GSCs in tumor tissue

Although CSCs have been identified as an important factor in tumor initiation and growth, their characteristics remain obscure concerning their heterogeneity. Here we found in our experiments that although seven of the nine human gliomas gave rise to proliferating tumor spheres, different numbers of spheres arisen from equal primary glioma cells among tumor samples. It should be noted that the specimens which did not give rise to proliferating neurospheres were patient #1 (oligoastrocytoma, gradeII) and #7 (anaplastic astrocytoma, grade III), with comparable tumor grades with the other specimens (Additional file 1: Table S1). Because samples are usually drawn from the periphery of the ablated tumor bulk, these two specimens might contain a certain amount of normal tissues. Overall, equal number of cells from high-grade and recurrent tumors, such as giant cell glioblastoma (WHO grade IV) and oligoastrocytoma (WHO grade III, recurrent tumor) often generate more primary tumor spheres. Due to limited number of samples, our accumulated results could not statistically lead to the conclusion that high-grade tumors contain more GSCs at present. However, the tendency described above indicated that the original frequency of GSCs might be different among samples according to tumor grades, or the GSCs from high-grade tumor tissues might show more typical properties of stem cells with higher proliferation and self-renewal ability.

### INP-like cells in GSCs population

In normal development of the brain, neurons and glia are generated from both NSCs and more limited INPs. And blockade of Notch signaling in NSCs have been shown to promote their conversion into INPs [20,21]. GSCs can differentiate into neurons and astrocytes in culture medium with serum, as shown by our results and previous studies [4]. Like INPs, it is possible that an intermediate glioma progenitor cells (IGPs) also exist, linking the GSCs-IGPs-Neuron/glia hierarchy in tumor microenvironment [32]. Our results show that blocking Notch signaling in the primary tumor spheres leads to down-regulated mRNA level of CD133, a well accepted marker of GSCs at present, indicating a decrease of GSCs. Simultaneously, the mRNA level of Hes5 and Glast, two markers highly expressed in NSCs were also decreased, while that of Mash1, a marker up-regulated in INPs was increased in primary tumor spheres after being treated with GSI [20]. In addition, Tubulin α1, an INP marker, seems can not distinguish IGPs from GSCs. Since GSI-treated primary tumor spheres could still gave rise to secondary spheres, unless much fewer than that derived from control primary spheres, the CD133^low^/GLAST^low^/Hes5^low^/Mash1^high ^IGPs might exist, with its number increased and proliferating ability decreased after the blockade of Notch signaling. Therefore, inhibiting Notch signaling might have therapeutic potential for human gliomas by exhausting GSCs and instruct them into less proliferative IGPs and differentiated neural cell types.

### Double positive cell types in the derivatives of GSCs

Although tumor-derived stem cells had many similarities to normal NSCs, it is important to note that differences might exist between them. Sphere differentiation assay on the specimen of 4# patient demonstrated that GSCs could give rise not only to neurons and glia but also to a few cells that expressed both Map2 and GFAP, the molecular markers of astrocyte and neuron, respectively (Figure S3). Previous studies have reported similar abnormal cells in culture derived from pediatric and adult brain tumors [4,33], indicating that such dual-fate cells might represent a significant fraction of GSCs derived progeny. These Map2^+^/GFAP^+ ^cells sometimes appeared larger than other cells derived from the same sphere (Figure S3). In addition, the GFAP positive glial cells derived from GSCs showed abnormal morphology, with slim cell bodies and neurites, compared with that derived from NSCs (Figure 3D, Figure 5E). Although morphological differences might exist between mouse and human glial cells, previous research on normal human tissue demonstrated that GFAP staining of human glial cells showed similar morphology with that of mouse glial cells [34]. Therefore, the morphological difference of GFAP positive glial cells might be attributed to whether they are NSC-derived or GSC-derived. Genetically, the generation of the double-positive cells and dysmorphic glial cells may accompany with gene mutation or abnormal activation of some signal pathways, leading to aberrant reprogramming procedure of GSCs, compared with normal differentiation of NSCs.

### GSI-resistance of GSCs at the early stage of GSI treatment

In our study, we found that the numbers of both primary and secondary tumor spheres were decreased in the long run (7-day culture) after GSI treatment compared with the controls. However, cell cycle analysis results showed that although Notch blockade significantly reduced the ratio of the G2+M phase in NSCs, there is no obvious effect on the percentage of proliferating GSCs within 72 h after GSI treatment. These results indicate that, compared with NSCs, another distinctive feature of GSCs was that the former are more sensitive to GSI, while the latter displays a certain degree of resistance to GSI treatment at the early stage of the treatment. Due to the limited amount of primary glioma specimens, the cell cycle analyses were executed on primary tumor spheres from three independent tumor samples. Therefore, the resistance to GSI in GSCs at the early stage of the cell cycle might be a general characteristic in gliomas, or it only represents a few cases of glioma patients which might display resistance in the preclinical trial of GSI treatment. Previous research show that treatment with dipeptide GSI resulted in a marked reduction in medulloblastoma growth [35]. More recently, a clinical trial for a Notch inhibitor, MK0752 (developed by Merck, Whitehouse Station, NJ), has been launched for T-cell acute lymphatic leukemia and breast cancer patients (http://www.clinicaltrials.gov/ct/show/NCT00100152). Although GSI seems to be a promising reagent targeting GSCs by interfering Notch signaling, our results suggested that its effect might be limited to some glioma patients. Therefore, drug combination should be used at the early stage of therapy. However, since our results are based on in vitro culture system of patient-derived samples, more accurate conclusion could be drawn from animal models or preclinical trials in future study.

### The mechanisms of Notch signaling in regulating the proliferation and differentiation of GSCs

The mechanistic links between Notch signaling and the proliferation and differentiation of GSCs were presumably governed by more than one mechanism. In our study, the decreased proliferation and increased differentiation of GSCs upon GSI treatment are accompanied with down-regulation of Hes1 and Hes5, the canonical Notch downstream effectors. In addition, the expression level of Mash1, a proneural gene antagonized by the Hes genes was up-regulated in GSI-treated primary tumor spheres. Therefore, the canonical Notch-CBF1-Hes axis seems also play critical roles in the proliferation and differentiation of GSCs, as its function in NSCs [11].

On the other hand, Notch signaling has been shown to have both negative and positive influences on cell cycle progression [11,36]. In the present study, we observed that the proliferation of GSCs decreased significantly in the long term culture, although GSI resistance of three glioma samples was present (see above). Mutations of p53, pTEN and H-Ras, have been identified in tumor tissues of giloma patients. And Notch signaling has been shown to crosstalk with p53 and pTEN signaling pathway, two major regulators of cell cycle [37,38]. In addition, down-regulation of Notch signaling in H-Ras-transformed human breast cells led to a significant decrease in their proliferation [39]. Therefore, how Notch signaling promotes the cell cycle of GSCs is yet to be explored, on the scenery of the complex signal crosstalk and genetic circuitry.

## Conclusion

Our data indicate that like NSCs, Notch signaling maintains the patient-derived GSCs by promoting their self-renewal and inhibiting their differentiation, and support that Notch signal inhibitor might be a prosperous candidate of the drug treatment targeting CSCs for gliomas, however, with GSI-resistance at the early stage of treatment.

## Competing interests

The authors declare that they have no competing interests.

## Authors' contributions

YYH and MHZ carried out tissue culture, animal experiments and gene expression analyses, participated in study design and manuscript preparation. GC and LLi carried out specimens collection. LLiang, FG and YNW helped histological examination and immunohistochemistry staining. LAF and HH designed the study and prepared the manuscript. All authors read and approved the final manuscript.

## Pre-publication history

The pre-publication history for this paper can be accessed here:

http://www.biomedcentral.com/1471-2407/11/82/prepub

## Supplementary Material

Additional file 1 **Hu et al Supplementary materials **The file contains Table S1-S3, Figure S1-S3 and their figure legends Click here for file
