# Supplementary material for: Notch signaling contributes to the maintenance of both normal neural stem cells and patient-derived glioma stem cells
Source: BMC Cancer. 2011 Feb 22;11:82. doi: 10.1186/1471-2407-11-82 (PMC3052197; doi:10.1186/1471-2407-11-82)
Supplement: Additional file 1 — Hu et al Supplementary materials The file contains Table S1-S3, Figure S1-S3 and their figure legends [file 1471-2407-11-82-S1.DOC]

**Supplemental Table 1**

Table S1. Patient population involved in this study.

| **Patient#** | **Sex** | **Age** | **Pathological subtype** | **Grade** |
| --- | --- | --- | --- | --- |
| 1 | F | 33 | oligoastrocytoma | Ⅱ |
| 2 | M | 35 | astrocytoma | Ⅲ |
| 3 | M | 48 | anaplastic oligodendroglioma | Ⅲ |
| 4 | F | 39 | anaplastic oligodendroglioma | Ⅲ |
| 5 | M | 43 | anaplastic oligoastrocytoma | Ⅲ |
| 6 | F | 44 | oligodendroglioma | Ⅱ |
| 7 | F | 28 | anaplastic astrocytoma | Ⅲ |
| 8 | M | 47 | giant cell glioblastoma | Ⅳ |
| 9 | M | 46 | anaplastic oligoastrocytoma | Ⅲ |

**Supplemental Table 2**

**Table S2. Number of patients in each experiment.**

| **assays** | **No. of the sample** |
| --- | --- |
| Identification of GSC | 2#, 3# |
| Proliferation of GSC | 2#, 3#, 4#, 5# |
| Self-renewal ability of GSC | 2#, 3#, 4#, 5# |
| Differentiation of GSC | 2#, 4#, 5# |
| Quantitative RT-PCR | 3#, 5#, 6#, 8#, 9# |
| DNA content analysis | 5#, 8#, 9# |

**Supplemental Table 3**

**Table S3**. Primers used in this study.

| Genes | Primers |
| --- | --- |
| Human-GAPDH | Sense: 5’-GCACCGTCAAGGCTGAGAAC-3’ |
|  | Antisense: 5’-TGGTGAAGACGCCAGTGGA-3’ |
| Human-CD133 | Sense: 5’-AGTGGCATCGTGCAAACCTG-3’ |
|  | Antisense: 5’-CTCCGAATCCATTCGACGATAGTA-3’ |
| Human-GLAST | Sense: 5’-AACGAAGCCATCATGAGACTGGTAG-3’ |
|  | Antisense: 5’–CACGGTGTACATGGCAAGCTG-3’ |
| Human-Mash1 | Sense: 5’- GTCACAAGTCAGCGCCCAAG-3’ |
|  | Antisense: 5’-CTGTAGCCAA AGCCGCTGAAG-3’ |
| Human-Tubulin α1 | Sense: 5’- TATGCCCGAGGGCACTACAC-3’ |
|  | Antisense: 5’-AAACCAAGAAGCCCTGGAGAC-3’ |
| Human-Hes5 | Sense: 5’-CACCAGGACTACAGCGAAGGCTA-3’ |
|  | Antisense: 5’-TGG AGCGTCAGGAACTGCAC-3’ |
| Human-Hes1 | Sense: 5’-TGGAAATGACAGTGAAGCACCTC-3’ |
|  | Antisense: 5’-TCGTTCATGCACTCGCTGAAG-3’ |
| Mouse-actin | Sense: 5’-CATCCGTAAAGACCTCTATGCCAAC-3’ |
|  | Antisense: 5’-ATGGAGCCACCGATCCACA-3’ |
| Mouse-GLAST | Sense: 5’-GAAGCCATCATGCGATTGGTC-3’ |
|  | Antisense: 5’-CACGGTGTACATGGCAAGCTG-3’ |
| Mouse-Mash1 | Sense: 5’-AAGAGCTGCTGGACTTTACCAACTG-3’ |
|  | Antisense: 5’-ATTTGACGTCGTTGGCGAGA-3’ |
| Mouse-Tubulin α1 | Sense: 5’-GTCCTGGACAGGATTCGCAAG-3’ |
|  | Antisense: 5’-GCTCAACCACAGCAGTGGAAAC-3’ |
| Mouse-Hes5 | Sense: 5’-GAAGGGCCGACATCCTGGAGA-3’ |
|  | Antisense: 5’-ACCAG-GAGTAGCCCTCGCTGTA-3’ |
| Mouse-Hes1 | Sense: 5’-AAAGACGGCCTCTGAGCAC-3’ |
|  | Antisense: 5’-GGTGCTTCACAGTCATTTCCA-3’ |

**Supplemental Figure S1**

**
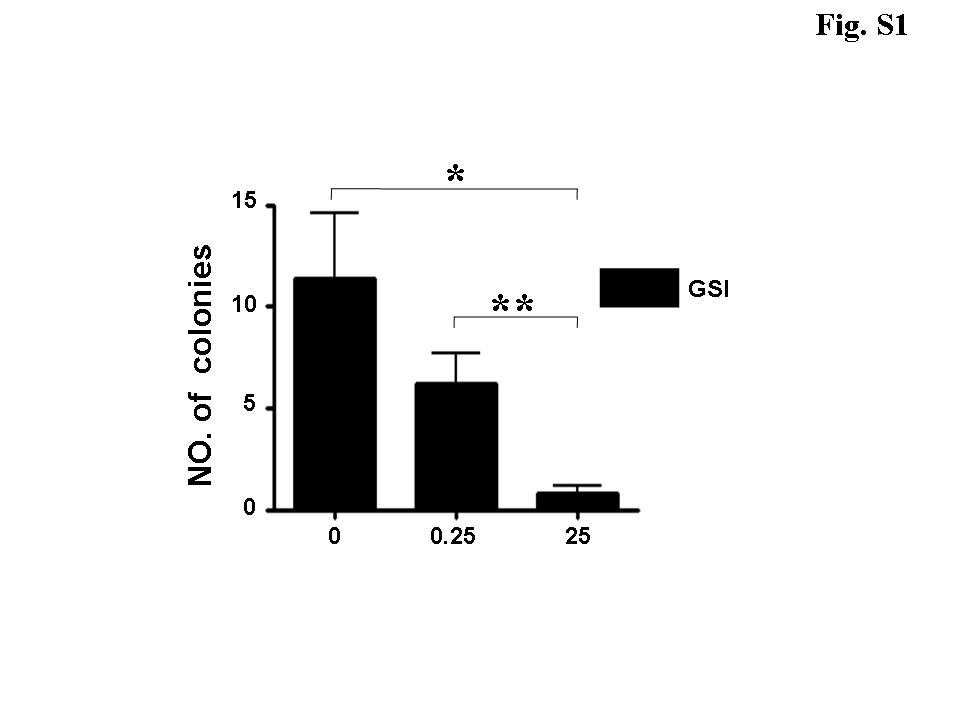
**

**GSI-treatments reduce the number of mouse primary neurospheres in a dose-dependent fashion.** Mouse embryonic brain-derived NSCs were cultured in the defined medium supplemented with GSI at 0 μmol/L, 0.25 μmol/L, 25 μmol/L. The number of colonies was counted 5 days after the start of the culture (n=3, 0 μmol/L, *P*=0.011, 0.25 μmol/L, *P*=0.007). *, *P*<0.05, **, *P*<0.01.

**Supplemental Figure S2**


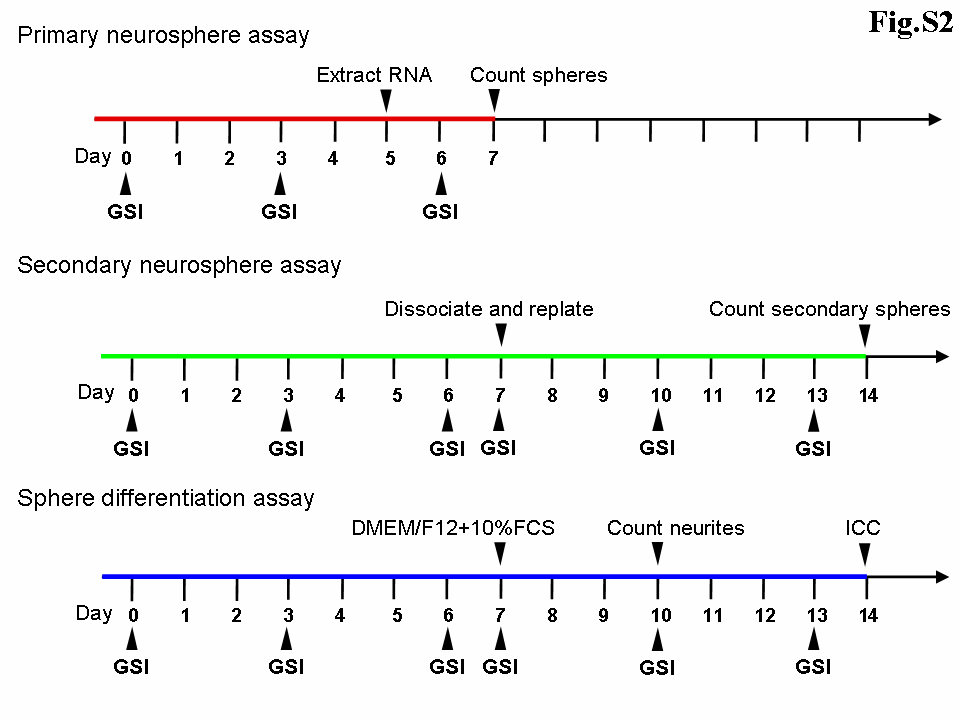


**Diagram of neurosphere assays.** Primary neurospheres were cultured for 7 days. Total RNA of neurospheres were extracted on the 5th day and the number of neurospheres were counted on the 7th day. For secondary neurosphere assay, primary neurospheres were dissociated and replated on the 7th day, and cultured for another week, the number of secondary neurospheres were counted on the 14th day of culture. For sphere differentiation assay, primary neurospheres were plated in differentiation conditional medium on the 7th day and culture for another week, the neurites of spheres were counted on the 10th day, and the immunocytochemistry (ICC) of differentiated neurospheres were performed on the 14th day. GSI or DMSO (not shown) were added every 3 days in culture, and were immediately added on the day of dissociation and replating.

**Supplemental Figure S3**

**
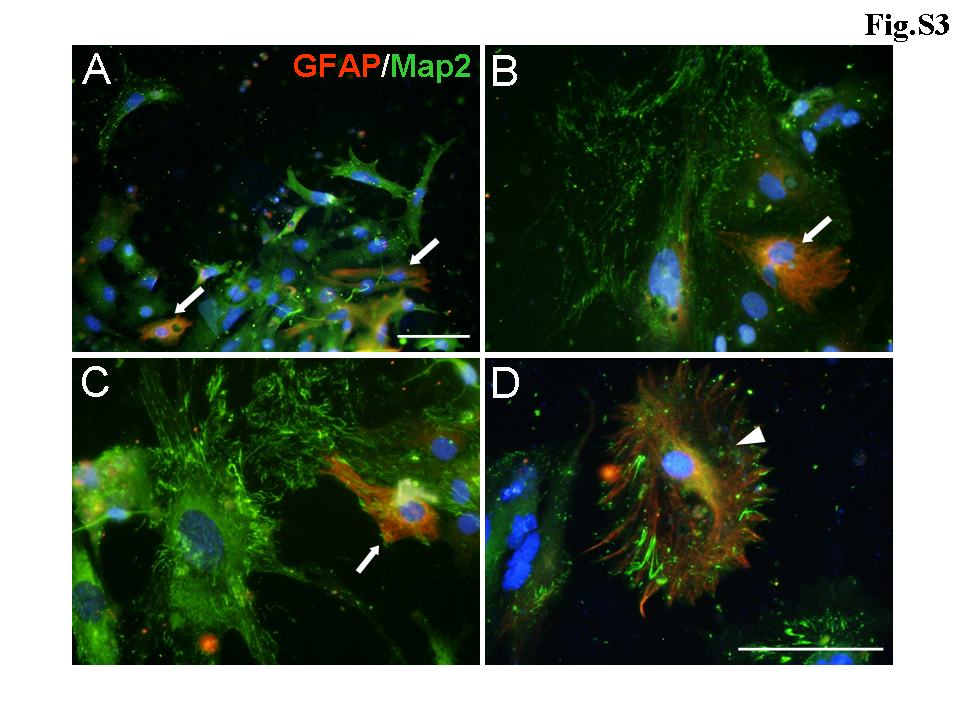
**

**Double positive cell types in the derivatives of patient-derived GSCs.** (A, B, C, D) A few cells derived from GSCs express both Map2 and GFAP (arrows in A, B, C), sometimes with larger cell bodies than other cells derived from the same sphere (arrowhead in D). Scale bar, 50 μm in A, and 50 μm in D for B-D.
